# Cooperative Effects in Weak Interactions: Enhancement of Tetrel Bonds by Intramolecular Hydrogen Bonds

**DOI:** 10.3390/molecules24020308

**Published:** 2019-01-16

**Authors:** Cristina Trujillo, Ibon Alkorta, José Elguero, Goar Sánchez-Sanz

**Affiliations:** 1 Trinity Biomedical Sciences Institute, School of Chemistry, The University of Dublin, Trinity College, Dublin 2, Ireland; trujillc@tcd.ie; 2 Instituto de Química Médica, CSIC, Juan de la Cierva, 3, E-28006 Madrid, Spain; ibon@iqm.csic.es (I.A.); iqmbe17@iqm.csic.es (J.E.); 3 Irish Centre of High-End Computing, Grand Canal Quay, Dublin 2, Ireland; 4 School of Chemistry, University College Dublin, Belfield, Dublin 4, Ireland

**Keywords:** non-covalent interactions, MP2, binding energy, intramolecular hydrogen bonds, tetrel bonds

## Abstract

A series of silyl and germanium complexes containing halogen atoms (fluorine and chlorine atoms) and exhibiting tetrel bonds with Lewis bases were analyzed by means of Møller-Plesset computational theory. Binding energies of germanium derivatives were more negative than silicon ones. Amongst the different Lewis bases utilized, ammonia produced the strongest tetrel bonded complexes in both Ge and Si cases, and substitution of the F atom by Cl led to stronger complexes with an ethylene backbone. However, with phenyl backbones, the fluorosilyl complexes were shown to be less stable than the chlorosilyl ones, but the opposite occurred for halogermanium complexes. In all the cases studied, the presence of a hydroxyl group enhanced the tetrel bond. That effect becomes more remarkable when an intramolecular hydrogen bond between the halogen and the hydrogen atom of the hydroxyl group takes places.

## 1. Introduction

One of the major achievements in contemporary chemistry was the introduction by Jean-Marie Lehn of supramolecular chemistry [1]. According to Vögtle “In contrast to molecular chemistry, which is predominantly based upon the covalent bonding of atoms, supramolecular chemistry is based upon intermolecular interactions, i.e., on the association of two or more building blocks, which are held together by intermolecular bonds”. Today, these intermolecular bonds are called weak interactions regardless of whether they are intra or intermolecular. There are numerous weak interactions, also known as non-covalent interactions, and they have been shown to be of the upmost importance across different domains including biology, chemistry and material science [2]. These non-covalent interactions has been categorised based on the interacting atoms involved: hydrogen bonds [3,4], halogen bonds [5], hydride bonds [6,7], pnictogen bonds [8,9,10,11,12,13], chalcogen interactions [14,15,16,17,18] and tetrel bonds [19,20,21,22]. The latter, tetrel bonds, are defined, analogous to halogen bonds, as interactions between electron donors and tetrel atoms (C, Si and Ge), in which the tetrel atom acts as an electron acceptor, usually through an electron-deficient outer lobe of a p orbital, called a σ-hole [23] by Politzer and Murray [24,25,26], which is formed in the tetrel atom, especially when the atom bonded to the tetrel is highly electronegative. It has also been shown that interactions through σ-holes are mainly driven by the electrostatic interaction term [27,28,29,30,31,32,33,34,35,36,37,38].

**Scheme 1:**
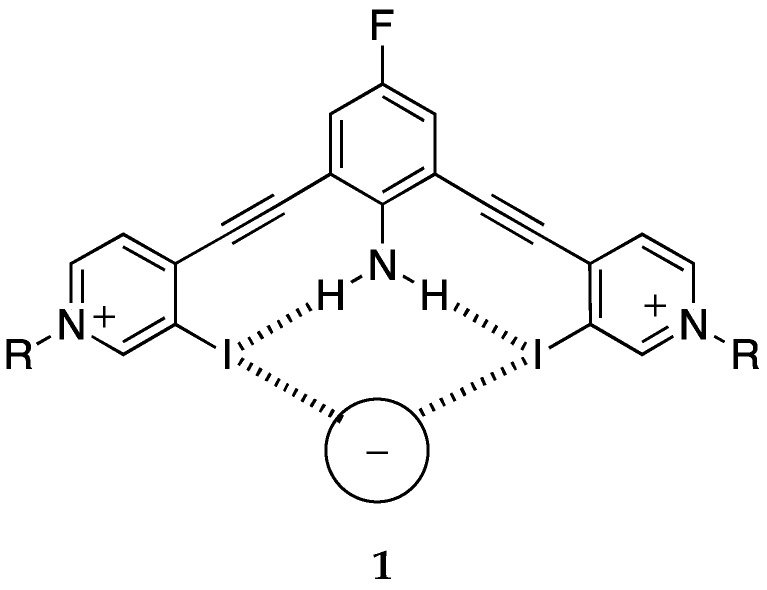
Schematic description of 2,6-bis(4-ethynylpyridinyl)-4-fluoroaniline interacting with anions.

Related to this topic is cooperativity, usually intermolecular (allosteric), but also, although much less studied, intramolecular [39]. There are two relevant papers. Berryman et al. reported the synthesis and anion-binding properties of receptor **1** (2,6-bis(4-ethynylpyridinyl)-4-fluoroaniline) which can act as a halogen donor, trapping a wide variety of anions (Cl^−^, Br^−^, I^−^, SCN^−^, NO_3_^−^, HSO_4_^−^, H_2_PO_4_^−^ and ReO_4_^−^) [40] (Scheme 1). The presence of intramolecular N–H···I hydrogen bonds (HBs) increases the strength of the I···anion halogen bonds (XBs).

**Scheme 2:**
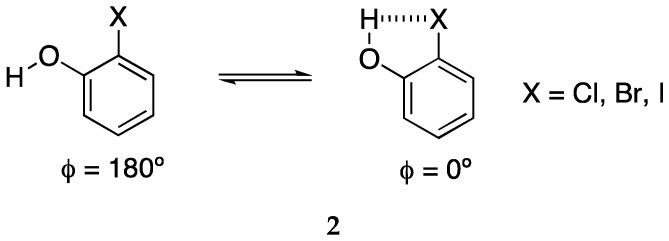
The two conformers of 2-halophenol.

Ho and co-workers described how the σ-hole responsible for the XB is substantially increased by an intramolecular HB [41]. The model they selected was 2-halophenol (**2**) that can exist as two limit conformations (Scheme 2), the *anti* one without HB and the *syn* one with an intramolecular HB (IMHB).

For each X, they calculated the electrostatic potential map as a function of the torsion angle ϕ. They found that the positive zone increases considerably when the torsion angle approaches 0°, i.e., when there is an IMHB.

**Scheme 3:**
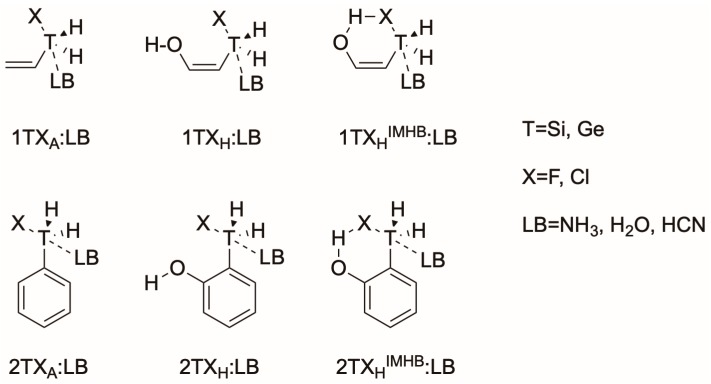
Schematic representation of the nTX:LB complexes subject to study.

In the present article, the effect of the intramolecular hydrogen bond in fluorosilyl and fluorogermanium derivatives and their abilities as tetrel bond donors is studied (Scheme 3). The following notation will be used to label the complexes studied throughout the manuscript: nTX_a_^Z^:LB, where “n” stands for the two types of carbon backbone (1=allyl, 2=phenyl), “T” stands for the tetrel atom (Si or Ge), “Z” indicates whether the complex exhibits, or does not exhibit, an intramolecular hydrogen bond (IMHB or none), “a” indicates the presence of an OH group in the molecule (hydroxyl=H) or not (allyl=A). Finally, LB stands for the Lewis Base involved (NH_3_, H_2_O or HCN). For example, 1GeCl_A_^IMBH^:NH_3_ corresponds to an allylchlorogermanium derivative interacting with ammonia in which the OH group is present and forming an intramolecular hydrogen bond. The short notation 1TX:LB will be used to refer the whole complex family. 

## 2. Results

In order to evaluate the appropriate computational level for the present study, a benchmark across different basis sets has been carried out. For such purpose, 1SiF:NH_3_ complexes (1SiF_A_:NH_3_, 1SiF_H_:NH_3_, and 1SiF_H_^IMHB^:NH_3_) were optimized by means of Møller-Plesset (MP2) using different basis sets, i.e., aug-cc-pVDZ (avdz), aug-cc-pVTZ (avtz), aug-cc-pVQZ (avqz), and using Helgaker’s method to extrapolate to the complete basis set (CBS) with two pairs of basis: aug-cc-pVDZ and aug-cc-pVTZ (CBS^DT^) and aug-cc-pVTZ and aug-cc-pVQZ (CBS^TQ^). Using those levels, the binding energies (Eb), obtained as a difference of the energy of the complex minus the energies of the isolated monomers in their optimized structure, for these three complexes, were calculated and are summarized in Table 1. As observed, for the evolution of the energy values, avdz < avtz < avqz, it seems that there is a strong dependency of the binding energy with respect to the basis set size. When extrapolation to the complete basis set (CBS) is taken into account, it was also observed that extrapolations using two different pairs of basis set, CBS^DT^ and CBS^TQ^, also provide different results, with the latter, in our opinion, more accurate than the former. Taken into account the outcome of the current benchmark and the computational feasibility of the calculations, the MP2/CBS^TQ^ computational method was chosen to evaluate the rest of the complexes, and from hence forth, named MP2/CBS for simplicity.

### 2.1. Allylfluorotetrel Derivatives: Effect of the Lewis Bases (1TF:LB)

**Figure 1:**
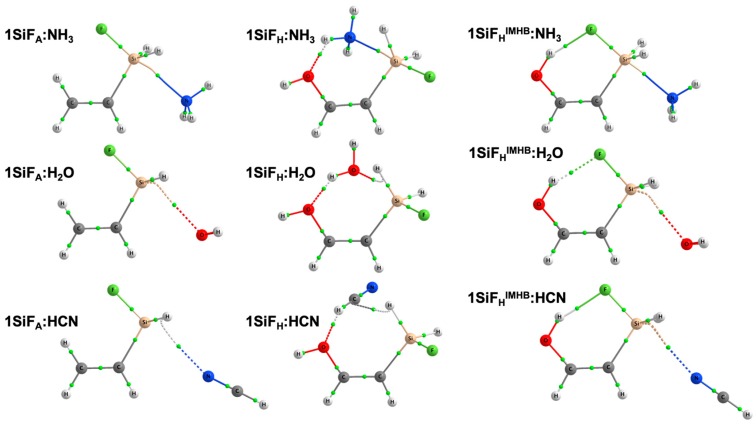
Molecular graphs for 1SiF:LB complexes at the MP2/aug-cc-pVTZ computational level. Green dots correspond to bond critical points.

The effect of the Lewis bases upon complexation within 1TF:LB complexes (Figure 1) was evaluated using three different Lewis bases, i.e., NH_3_, H_2_O and HCN. The intermolecular T···Y (Y = N or O) distances at the MP2/aug-cc-pVTZ computational level are gathered in Table 2. Molecular graphs and Cartesian coordinates can be found in the electronic supplementary information (Table S1). The intermolecular Si···N distances found in 1SiF:NH_3_ range from 2.276 (1SiF_H_^IMHB^:NH_3_) to 2.518 Å (1SiF_A_:NH_3_), while in 1GeF:NH_3_, the Ge···Y distance ranges from 2.450 (1GeF_H_^IMHB^:NH_3_) to 2.661 Å (1GeF_A_:NH_3_). As observed, 1TF_H_^IMHB^:NH_3_ complexes present the shortest intermolecular T···Y distances (Table 2). Looking at the 1SiF_A_:NH_3_ complex, the intermolecular Si···N distance is 2.518 Å. When the 1SiF_H_:NH_3_ complex is taken into account, there is a shortening of 0.183 Å on the Si···N distance. This shortening can point to a decrease in the electron density on the Si atom due to the OH group and therefore an increase in the σ-hole depth, which eventually will enhance the tetrel bond. This shortening is even more visible in 1SiF_H_^IMHB^:NH_3_ complex (0.242 Å), which may indicate that the intramolecular hydrogen bond (IMHB) enhances the tetrel bond. In the case of 1GeF:LB germanium complexes, similar but more pronounced trends in the intermolecular Ge···N distances were found due to the IMHB and hydroxyl groups. Furthermore, the T-F distance was also analyzed to evaluate the polarity of the T-F bonds with the IMHB. T-F distance for the 1SiF_A_:NH_3_ complex is 1.643 Å, while in 1SiF_H_:NH_3_ and 1SiF_H_^IMHB^:NH_3_ complexes, they are 1.654 and 1.685 Å, respectively, indicating an increase in the charge transfer to the T-F σ*T-F antibonding orbital, particularly in the complexes exhibiting IMBH. The same is true for 1GeF:NH_3_ complexes (1.768, 1.776 and 1.815 Å for 1GeF_A_:NH_3_ complex, 1GeF_H_:NH_3_ and 1GeF_H_^IMHB^:NH_3_ complexes, respectively).

When different Lewis bases are considered (Table 2), it can be seen that complexes with ammonia have the shortest intermolecular T···Y distances. However, similar trends were observed for 1TF:H_2_O complexes compared with 1TF:NH_3_ complexes, where the T···Y distance evolves as follows: 1TF_A_:H_2_O > 1TF_H_:H_2_O > 1TF^IMHB^_A_:H_2_O. In the case of 1TF:HCN complexes, the 1TF_H_:HCN complex seems to deviate from this trend, exhibiting a lengthening in the intermolecular T···Y distance. The reason behind is that in those complexes (both for 1SiF_H_:HCN and 1GeF_H_:HCN), the O atom from the OH group acts as an electron donor and performs an intermolecular hydrogen bond with the Lewis base. In other words, the Lewis base acts as a hydrogen donor. That provokes a re-orientation of the Lewis base which lengthens the T···N distance. If the molecule is constrained to Cs symmetry, forcing those abovementioned to be co-planar, an imaginary frequency is found which, if followed, reverts into the actual rotated structure with *C*_1_ symmetry.

In order to evaluate the interaction energies between 1TF compounds and the Lewis base, binding energies (E_b_), interaction energies (E_int_) and deformation energies (E_def_) were calculated at the MP2/CBS computational level and are summarized in Table 3 and with a histogram plotted in Figure 1. Binding energies (E_b_) and interaction energies (E_int_) were calculated as described in the “Materials and Methods” section. The deformation energy (E_def_), also called re-organization energy, was calculated as the difference between E_b_ and E_int_. E_b_ ranges from −21.8 to −41.0 kJ·mol^−1^ for 1TF:NH_3_ complexes. In 1TF:H_2_O, it ranges from −15.9 to −26.9 kJ·mol^−1^, and −14.5 to −25.1 kJ·mol^−1^ for 1TF:HCN ones. In all cases, 1TF:NH_3_ complexes show stronger interactions than 1TF:H_2_O or 1TF:HCN ones as per the E_b_ values. Focusing on the 1SiF:NH_3_ complexes, it was observed that the E_b_ value calculated for 1SiF_A_:NH_3_ is −21.8 kJ·mol^−1^, and when the hydroxyl groups are present, E_b_ becomes slightly more negative (−22.9 kJ·mol^−1^), which indicates a strengthening of the interaction. This strengthening is even larger in 1SiF_H_^IMHB^:NH_3_ (E_b_ = −31.7 kJ·mol^−1^), which shows that when the IMHB takes place, the interactions between the complex and the Lewis base are stronger. The same occurs for the 1GeF:NH_3_ complexes. In the case of water complexes, the E_b_ follows the same trend as in ammonia complexes. However, the 1TF_H_:H_2_O complex presents slightly more negative values of E_b_ than the 1TF_H_^IMHB^:H_2_O complex, which is likely due to a secondary intermolecular hydrogen bond in which water is acting as a hydrogen bond donor (Figure 1). This could be the reason behind the extra stabilization in the E_b_. The opposite happens for the 1TF:HCN complexes in which 1TF_H_:HCN is less stable than its parental complex, 1TF_A_:HCN. As explained above, this is provoked by a re-orientation of the Lewis base in the 1TF_H_:HCN due to the presence of the hydroxyl group, which acts as an electron donor and forms an intermolecular hydrogen bond with the HCN (Figure 1). Poor correlations between the binding energy and the intermolecular distances were found, which may be due to the secondary hydrogen bonds in the 1TF_H_:LB complexes and electronic repulsions between atoms due to the electron lone pairs [42]. 

**Figure 2:**
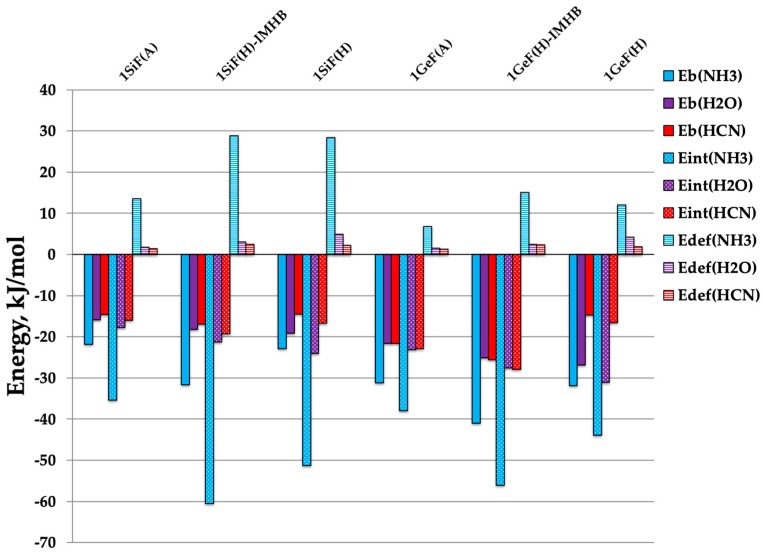
Histogram for the different energies: E_b_ (solid), E_int_ (dotted) and E_def_ (stripped) for each Lewis base considered: NH_3_ (**blue**), H_2_O (**purple**) and HCN (**red**).

To evaluate the re-organization energy, i.e., the relaxation energy of the monomers and the energy penalty upon complexation, the interaction energies were evaluated. As observed (Figure 2), E_int_ values for all the complexes studied are more negative than the corresponding E_b_ values; this is particularly dramatic for the 1SiF:NH_3_ complexes in which the E_int_ values are the most negative of all complexes. When the difference of both quantities (E_b_ − E_int_) is taken into account, the deformation energy, E_def_, values (Table 3) indicate that 1TF:NH_3_ complexes suffer a larger penalty in binding energy due to re-organization than 1TF:H_2_O and 1TF:HCN complexes.

### 2.2. Allylhalotetrel Derivatives: Effect of the Halogen (1TX:NH_3_)

Once the effect of the Lewis base on the tetrel interaction and the relative energy between the three different complexes was determined, the effect of substituting the halogen atom (X) bonded to the tetrel atom (T=Si or Ge) was studied. For this purpose, and keeping the same Lewis base (NH_3_), fluorine atom was replaced by a chlorine atom within the 1TX:NH3 complexes. Intermolecular T···N distances are summarized in Table 4. The shortest T···N distance corresponds to the 1SiF_H_^IMHB^:NH_3_ complex (2.276 Å) while the largest is found in the 1GeCl_A_:NH_3_ complex (2.767 Å). As observed in Table 4, 1TF:NH_3_ complexes exhibit shorter intermolecular T···Y distances than 1TCl:NH_3_ ones, with the exception of the 1SiF_H_:NH_3_ complex, which presents a slightly longer (0.044 Å) T···N distance than the 1SiCl_H_:NH_3_ complex. Furthermore, the T-F distances found for 1TF:NH_3_ complexes suggest an increase in the polarity of the T-F bond, and a similar phenomenon is observed for 1TCl:NH_3_ (Table 4) in which the T-Cl bond distances exhibit a lengthening in 1TX_H_:NH_3_ and 1TX_H_^IMHB^:NH_3_ complexes with respect to those in 1TX_A_:NH_3_ complexes.

In terms of binding energies, 1TF:NH_3_ complexes show slightly more negative E_b_ values than 1TCl:NH_3_ complexes, which may be caused by an increase in the depth of the σ-hole on the tetrel atom (Table S2). Fluorine atoms withdraw more electron density, therefore the σ-hole on the tetrel atom is deeper, so 1TF:NH_3_ complexes will have more negative E_b_ values than 1TCl:NH_3_ complexes. Once again, the only exception corresponds to 1SiCl_H_:NH_3_ with an E_b_ value of −24.5 kJ·mol^−1^, while the 1SiF_H_:NH_3_ E_b_ value is −22.9 kJ·mol^−1^. In both cases, the halogen atom does not form any IMHBs, which reinforces the idea that IMHBs are stabilizing the complex. Regarding the interaction energy, E_int_ is shown to be more negative than E_b_, which is as expected because the E_b_ suffers from the re-organization penalty. In fact, E_def_ values indicate that this penalty is larger for silyl complexes than germanium ones, and within the same tetrel family (1TX:NH_3_), the deformation energy is much larger, up to three times larger in some cases, for complexes with hydroxyl groups (1TF_H_:NH_3_ and 1TX_H_^IMHB^:NH_3_) than in their parental complexes (1SiX_A_:NH_3_).

### 2.3. Phenyl Halogen Tetrel Derivatives: Effect of the Backbone (2TX:NH_3_)

Finally, different carbon backbones have been evaluated by means of replacing the allyl backbone with a phenyl ring focusing only on complexes with ammonia. The intermolecular T···N distances, E_b_, E_int_, E_def_ and T-X bond distances are summarized in Table 5. Intermolecular T···N distances in 2TX:NH_3_ complexes present similar values to those in 1TX:NH_3_ complexes, but with slight variations. 2TF_A_:NH_3_ complexes (both for Si and Ge) exhibit longer T···N distances (2.596 and 2.703 Å, respectively) than 1TF_A_:NH_3_ complexes (2.518 and 2.661 Å), and the same occurs for 2TX_H_^IMHB^:NH_3_ complexes, while the opposite is true for 2TF_H_:NH_3_ (Si = 2.330 and Ge = 2.553 Å) compared with 1TF_H_:NH_3_ (Si = 2.335 and Ge = 2.570 Å). As in 1TX:NH_3_ complexes, the ones with hydroxyl groups present, both 2TX_H_:NH_3_ and 2TX_H_^IMHB^:NH_3_ exhibit a shortening of the T···N distances. However, looking at the T-X bond distances, both for X=F and Cl, a lengthening is observed in 2TX_H_:NH_3_ and 2TX_H_^IMHB^:NH_3_ consistent with the results mentioned above and the increase in polarity of the T-X bond with the presence of hydroxyl groups and IMHBs.

Binding energies for 2TX:NH_3_ complexes are found to be within the same range as 1TX:NH_3_ complexes, with 2TX_A_:NH_3_ and 2TX_H_^IMHB^:NH_3_ complexes exhibiting larger E_b_ values than their corresponding allyl counterparts, while 2TX_H_:NH_3_ complexes present slightly smaller E_b_ values than 1TX_H_:NH_3_ complexes, both for T=Si, Ge and X=F, Cl. Eb values show that in all cases, 2SiCl:NH_3_ complexes have a stronger interaction with NH_3_ than 2SiF:NH_3_ complexes, while the opposite is true for the germanium derivatives. In terms of interaction energies, E_int_, values in Table 5 reveal similar features to those found for the allyl complexes, i.e., large negative values of E_int_, twice as much in some cases (for example: 2SiX_H_:NH_3_). This indicates a substantial re-organization energy, which was also confirmed when the E_def_ values were analyzed. E_def_ values are very large, in fact, silyl complexes with hydroxyl groups (2SX_H_:NH_3_) present the largest deformation energies, as occurred in 1SX_H_:NH_3_ complexes. This can be explained in terms of electronic repulsion between groups, as explained above [42].

If we compare the evolution of the binding energy across the 2TX:NH_3_ complexes, it is seen that the presence of hydroxyl (2TX_H_:NH_3_) enhances the interaction with the Lewis base in about 10.2% (2SiF_H_:NH_3_) and 10.7% (2GeF_H_:NH_3_). This enhancement is even larger within 2TCl:NH_3_ complexes (33.4% and 12.4% in 2SiCl_H_:NH_3_ and 2GeCl_H_:NH_3_, respectively). Besides, the enhancement of the binding energy reaches the maximum value when the IMHB takes place in the complex, reaching up to 38.2% of enhancement on the E_b_ with respect to the 2TX_A_:NH_3_ complex. 

### 2.4. Electron Density Properties

The electron density properties of the intermolecular tetrel interactions were studied by means of atoms in molecules (AIM) theory. In most of the cases, the tetrel bond is characterized by the existence of a bond critical point (BCP) between the tetrel atom and the electron donor on the Lewis base moiety (N or O) (Table S1). As it is denoted in the literature, this should be taken carefully, since the opposite is not true, i.e., the absence of a BCP between two interacting atoms does not necessary imply the absence of interactions [43]. It has been found that in some cases dominant weak interactions exhibit neither BCP nor bond paths [44]. However, while the BCPs should be taken cautiously and there is a huge debate about the interpretation of the bond paths and their relationship to the chemical bond, the existence of BCPs and their associated properties has been proven to be a useful tool to identify non-covalent interactions across the different varieties: hydrogen [45,46], halogen [47], pnicogen [48], chalcogen [14] and tetrel bonds [49].

The electron density parameters obtained are summarized in Table S3. Values of the electron density at the bond critical (ρ_BCP_) for 1SiF:NH_3_ and 1GeF:NH_3_ complexes range from 0.0270 to 0.045 a.u. and 0.0241 to 0.0366 a.u., respectively. Values for 1SiF:HCN and 1GeF:HCN complexes, which show similar T···N interactions, are smaller at 0.0101–0.0129 a.u. and 0.0132–0.0158 a.u., respectively. This is aligned with the interaction and binding energy values found and the intermolecular T···N distance evolution. Similar comparison trends are found for the Laplacian (∇^2^ρ _BCP_) values and total electron energy density (H_BCP_) values between those complexes.

1TCl:NH_3_ complexes show very similar ρ_BCP_ values to 1TF:NH_3_, concomitantly with similar Laplacian and H_BCP_ values, with slight differences. Cremer et al. demonstrated that the sign of the total electron energy density (H_BCP_), defined as the sum of G_BCP_ + V_BCP_, could be used to indicate the degree of covalency for chemical interactions [50,51,52]. H_BCP_ values have been found to be negative for interactions that significantly share electrons. In fact, in other interactions, such as pnicogen bonds, the electron density increment within intermonomeric regions has been postulated as a stabilizing factor [53]. As observed for 1TX:NH_3_ complexes (Table S3), negative H_BCP_ values are found and they may indicate a certain covalent character of the tetrel interaction. Furthermore, 1TX_H_^IMHB^:NH_3_ complexes present the most negative H_BCP_ values, which reinforces the idea that hydroxyl groups performing IMHB enhance tetrel bonds. 

**Figure 3:**
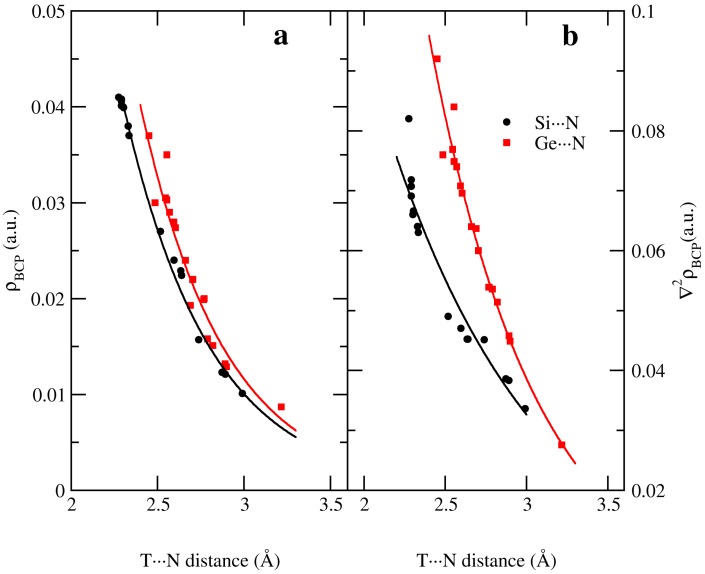
Exponential relationships between (**a**) electron density at the bond critical point and (**b**) Laplacian at the bond critical point with the intermolecular T···N distance at the MP2/aug-cc-pVTZ computational level.

Exponential relationships have been found between the electron density at the BCP and the intermolecular T···N distance; Si···N distances show better correlations (ρ = 3.8959 e^−1.9863 d(T···N)^, R^2^ = 0.991) than Ge···N (ρ = 5.7894 e^−2.0706 d(T···N)^, R^2^ = 0.943) interactions as observed in Figure 3a. Similar relationships have also been found between the Laplacian and T···N distance (∇^2^ρ = 0.76454 e^−1.0514 d(T···N)^, R^2^ = 0.947 and ∇^2^ρ = 3.6611 e^−1.5177 d(T···N)^, R^2^ = 0.982 for T= Si and Ge complexes, respectively) as depicted in Figure 3b.

**Figure 4:**
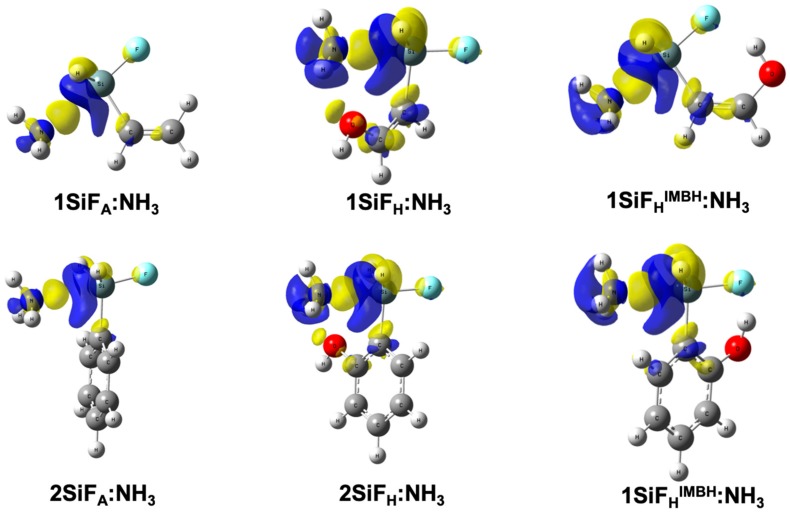
Electron density shift maps for the nSiF:NH_3_ (n = 1, 2) complexes on the ±0.002 a.u. electron density isosurfaces at the MP2/aug-cc-pVTZ computational level. Blue and yellow areas correspond to regions with a decrease and increase in electron density, respectively.

In order to provide further insight into the electron density changes upon complexation, electron density shift maps (EDS) at the ±0.002 a.u. isosurfaces are plotted in Figure 4. Blue areas correspond to those regions with a decrease in the electron density upon complexation, while yellow areas indicate those regions in which an increase in the electron density occurs. As observed, the 1SiF_A_:NH_3_ complex shows an increase (positive yellow area) in electron density between the N and Si atoms, corresponding to the donation of electron density from the Lewis base into the Si σ-hole. This pattern of depletion–increase in electron density has been observed for a wide range of inter/intramolecular interactions [14,54,55,56,57,58]. When 1SiF_H_:NH_3_ and 1SiF_H_^IMHB^:NH_3_ complexes are considered, the changes in electron density upon complexation become even more evident: blue (negative) and yellow (positive) areas are larger for 1SiF_H_:NH_3_ and 1SiF_H_^IMHB^:NH_3_ complexes than for 1SiF_A_:NH_3_ complexes, particularly in the areas surrounding the ammonia in which a drastic decrease in electron density is observed. This decrease is due to the larger electron density donation from the Lewis base in complexes with hydroxyl groups (1SiF_H_:NH_3_ and 1SiF_H_^IMHB^:NH_3_) than in the 1SiF_A_:NH_3_ one. When the phenyl backboned complexes are taken into account, the electron density shift maps show very similar increase/decrease patterns compared to the allyl one. Furthermore, there are no appreciable changes in the shape or size of the blue and yellow areas in comparison with the 1SiF:NH_3_ complexes, which is in consonance with the binding/interaction energies and the evolution of intermolecular T···N distances.

## 3. Materials and Methods

The structures of the complexes were optimized at the Møller-Plesset (MP2) [59]/aug-cc-pVTZ [60,61] computational level. Harmonic vibrational frequencies were computed at the same level used for the geometry optimizations in order to confirm that the stationary points are local minima. Calculations were performed using the Gaussian09 program [62]. Binding energies (E_b_) were calculated as a difference in the energy of the complex minus the energy of each monomer in their optimized geometry. Interaction energies (E_int_) were calculated as a difference in the energy of the complex minus the energy of each monomer, keeping the monomer geometry fixed in the complex optimized geometry. Finally, the deformation energy (E_def_), also called the re-organization energy, was calculated as the difference between E_b_ and E_int_. In order to provide more accurate energies the interaction energies were also estimated at the MP2/CBS (complete basis set) limit using the method of Helgaker et al. [63,64] from the calculated interaction energies with the aug-cc-pVTZ and aug-cc-pVQZ basis sets:

(1) $$E_{X}^{HF}=E_{CBS}^{HF}+Ae^{-\propto X}$$

(2) $$E_{X}^{MP2}=E_{CBS}^{MP2}+BX^{-3}$$

where *E*_X_ and *E*_CBS_ are the energies for the basis set with the largest angular momentum *X* and for the complete basis set, respectively.

The atoms in molecules (AIM) methodology [65,66] was used to analyze the electron density of the systems with the AIMAll program [67].

The intermolecular electron density shift (EDS) [58] was calculated using Equation (3):

EDS = ρ(nTX:NH_3_) − ρ(nTX) − ρ(NH_3_) (3)

where ρ(nTX:NH_3_), ρ(nTX) and ρ(NH_3_) stand for the electron density of the complex and both fragments in the geometry of the complex, respectively.

## 4. Conclusions

The complexation of a series of silyl (nSiX:LB) and germanium (nGeX:LB) complexes, with two different halogen atoms (X=F and Cl) was studied. Three different Lewis bases (NH_3_, H_2_O and HCN) were considered to form the complexes and the effect of the existence or absence of intramolecular hydrogen bonds on the intermolecular tetrel bond were analyzed.

Calculated geometrical parameters and binding and interaction energies suggested that the presence of hydroxyl groups enhances the intermolecular tetrel bond. In general, nTX_H_:LB and nTX_H_^IMHB^:LB complexes presented shorter intermolecular T···Y distances than their parental nTX_A_:LB complexes. This fact, concomitantly with the E_b_ values found: E_b_(nTX_H_^IMHB^:LB) < E_b_(nTX_A_:LB), indicates a strengthening of the tetrel bond with the presence of hydroxyl groups, whether it is forming an IMHB or not. However, the data analyzed reveal that when the IMHB takes place, the enhancement is even larger than without it.

Regarding the Lewis base, complexes with NH_3_ showed the most negative binding energies, followed by H_2_O and HCN.

The substitution of the fluorine atom (X=F) by a chlorine atom, in general, weakens the tetrel bond in allyl complexes (1TCl:NH_3_), which show E_b_ and E_int_ values larger than its fluorine counterparts. 

Finally, the effect of the carbon backbone was evaluated using two different backbones, allyl and phenyl. In spite of some differences, the intermolecular T···N distances and binding energies remain within the same range values.

## Figures and Tables

**Table 1:** Binding energies (E_b_), in kJ·mol^−1^, of 1SiF:NH_3_ complexes at the MP2 computational level with different basis sets including extrapolation to the complete basis set limit (CBS) with two different basis set pairs.

	avdz ^a^	avtz ^a^	avqz ^a^	CBS^DT b^	CBS^TQ b^
**1SiFA:NH3**	−29.3	−25.4	−23.8	−24.5	−21.8
**1SiFH:NH3**	−32.3	−28.2	−25.9	−27.4	−22.9
**1SiFHIMHB:NH3**	−40.5	−36.3	−34.4	−35.4	−31.7

^a^ avdz, avtz and avqz stand for aug-cc-pVDZ, aug-cc-pVTZ and aug-cc-pVQZ respectively. ^b^ CBS stands for extrapolation to the complete basis set limit, and DT and TQ refer to the pair of basis set used to extrapolate: avdz-avtz and avtz-avqz, respectively.

**Table 2:** Intermolecular distances, T···N(O) (T=Si, Ge) in Å for 1TF:LB complexes at the MP2/aug-cc-pVTZ computational level.

Comp.	NH_3_	H_2_O	HCN
1SiF_A_:LB	2.518	2.885	2.991
1SiF_H_:LB	2.335	2.883	4.033
1SiF_H_^IMHB^:LB	2.276	2.765	2.873
1GeF_A_:LB	2.661	2.829	2.898
1GeF_H_:LB	2.570	2.782	2.891
1GeF_H_^IMHB^:LB	2.450	2.720	2.789

**Table 3:** Binding energies (E_b_), interaction energies (E_int_) and deformation energies (E_def_) for the 1TF:LB complexes at the MP2/CBS computational level, in kJ·mol^−1^.

Complex	E_b_			E_int_			E_def_		
	NH_3_	H_2_O	HCN	NH_3_	H_2_O	HCN	NH_3_	H_2_O	HCN
1SiF_A_:NH_3_	−21.8	−15.9	−14.6	−35.2	−17.7	−16.1	13.6	1.8	1.4
1SiF_H_:NH_3_	−22.9	−19.2	−14.5	−51.3	−24.1	−16.7	28.4	4.9	2.2
1SiF_H_^IMHB^:NH_3_	−31.7	−18.2	−16.9	−60.6	−21.2	−19.2	28.8	3.0	2.4
1GeF_A_:NH_3_	−31.2	−21.6	−21.6	−38.0	−23.1	−22.9	6.8	1.5	1.3
1GeF_H_:NH_3_	−31.9	−26.9	−14.7	−43.9	−31.1	−16.6	12.0	4.2	1.9
1GeF_H_^IMHB^:NH_3_	−41.0	−25.1	−25.6	−56.1	−27.6	−27.9	15.1	2.4	2.3

**Table 4:** Binding energies (E_b_), interaction energies (E_int_) and deformation energies (E_def_), at the MP2/CBS computational level, in kJ·mol^−1^, intermolecular T···N distances and T-X bond distances (T=Si, Ge and X=F, Cl), in Å for 1TX:NH_3_ complexes at the MP2/aug-cc-pVTZ computational level.

	E_b_		E_int_		E_def_		T···N		T-X	
**Complex**	X=F	X=Cl	X=F	X=Cl	X=F	X=Cl	X=F	X=Cl	X=F	X=Cl
**1SiX_A_:NH_3_**	−21.8	−20.1	−35.4	−30.5	13.6	10.4	2.518	2.640	1.643	2.110
**1SiX_H_:NH_3_**	−22.9	−24.5	−51.3	−58.5	28.4	34.1	2.335	2.291	1.654	2.156
**1SiX_H_^IMHB^:NH_3_**	−31.7	−27.5	−60.6	−58.9	28.8	31.4	2.276	2.291	1.685	2.185
**1GeX_A_:NH_3_**	−31.2	−28.2	−38.0	−32.7	6.8	4.6	2.661	2.767	1.768	2.193
**1GeX_H_:NH_3_**	−31.9	−31.6	−43.9	−42.3	12.0	10.7	2.570	2.603	1.776	2.217
**1GeX_H_^IMHB^:NH_3_**	−41.0	−34.8	−56.1	−46.6	15.1	11.8	2.450	2.545	1.815	2.251

**Table 5:** Binding energies (E_b_), interaction energies (E_int_) and deformation energies (E_def_), at the MP2/CBS computational level, in kJ·mol^−1^, intermolecular T···N distances and T-X bond distances (T=Si, Ge and X=F, Cl), in Å for 2TX:NH_3_ complexes at the MP2/aug-cc-pVTZ computational level.

	E_b_		E_int_		E_def_		T···N		T-X	
**Complex**	X=F	X=Cl	X=F	X=Cl	X=F	X=Cl	X=F	X=Cl	X=F	X=Cl
**2SiX_A_:NH_3_**	−21.5	−21.5	−30.99	−30.78	9.5	9.2	2.596	2.635	1.638	2.111
**2SiX_H_:NH_3_**	−23.7	−28.7	−59.13	−61.07	33.9	33.2	2.330	2.305	1.652	2.150
**2SiX_H_^IMHB^:NH_3_**	−25.2	−27.9	−55.27	−59.79	31.6	31.1	2.301	2.291	1.677	2.176
**2GeX_A_:NH_3_**	−29.0	−27.8	−34.45	−31.73	5.4	3.9	2.703	2.771	1.766	2.194
**2GeX_H_:NH_3_**	−32.1	−31.3	−55.44	−48.18	15.3	13.3	2.553	2.594	1.776	2.214
**2GeX_H_^IMHB^:NH_3_**	−40.1	−34.9	−47.60	−44.99	15.5	13.7	2.485	2.554	1.809	2.244

## Supplementary Materials

The supplementary materials are available online.
