# Supplementary material for: Cooperative Effects in Weak Interactions: Enhancement of Tetrel Bonds by Intramolecular Hydrogen Bonds
Source: Molecules. 2019 Jan 16;24(2):308. doi: 10.3390/molecules24020308 (PMC6359400; doi:10.3390/molecules24020308)
Supplement: Supplementary file 1 [file molecules-24-00308-s001.pdf]

Supplementary Information

Cooperative effects in weak interactions: enhancement of tetrel bonds by intramolecular hydrogen bonds

Cristina Trujillo[1], Ibón Alkorta [2], José Elguero[2] and Goar Sánchez-Sanz[3,*]

[1] Trinity Biomedical Sciences Institute, School of Chemistry, The University of Dublin, Trinity College, Dublin 2, Ireland; trujillc@tcd.ie
[2] Instituto de Química Médica, CSIC, Juan de la Cierva, 3, E-28006 Madrid, Spain; ibon@iqm.csic.es; iqmb17@iqm.csic.es
[3] Irish Centre of High-End Computing, Grand Canal Quay, Dublin 2, Ireland & School of Chemistry, University College Dublin, Belfield, Dublin 4, Ireland; goar.sanchez@ichec.ie
* Correspondence: goar.sanchez@ichec.ie; Tel.: +353 1 5241608 (ext. 47)

- Table S1: Molecular graphs and Cartesian coordinates for all the complexes studied at the MP2/aug-cc-pVTZ computational level
- Table S2. Molecular electrostatic potential maxima corresponding to the σ-hole on the tetrel atom, in kcal/mol, on the 0.001 a.u. electron density isosuface at the MP2/aug-cc-pVTZ computational level.
- Table S3: electron density, Laplacian, and total energy density, H at the bond critical point, in a.u. and intermolecular distance, in Å, at the MP2/aug-cc-pVTZ computational level.

Table S1. Molecular graphs and Cartesian coordinates for all the complexes studied at the MP2/aug-cc-pVTZ computational level.

1T											
1SiF: NH_3				1SiF$^{OH}_{rot}$:NH_3				1SiF^{OH}:NH_3			
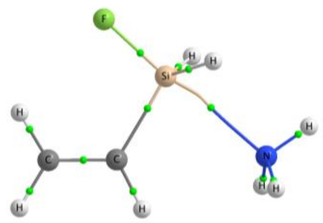				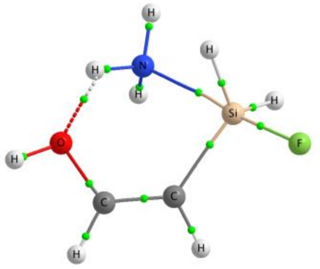				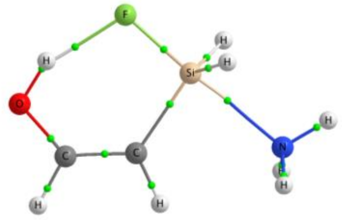			
C	1.107413	-0.757414	0.000000	C	-0.409368	-1.033608	-0.924454	C	-0.409368	-1.033608	-0.924454
C	1.817105	0.381775	0.000000	C	-0.116385	-1.716378	0.185628	C	-0.116385	-1.716378	0.185628
H	1.663940	-1.688472	0.000000	H	-0.551089	-1.665352	-1.794881	H	-0.551089	-1.665352	-1.794881
H	2.900810	0.378457	0.000000	H	-0.053085	-2.798940	0.216509	H	-0.053085	-2.798940	0.216509
H	1.325435	1.345784	0.000000	Si	-0.410071	0.817456	-1.212580	Si	-0.410071	0.817456	-1.212580
Si	-0.763743	-0.728934	0.000000	H	0.363931	1.661262	-0.282070	H	0.363931	1.661262	-0.282070
H	-1.428628	-1.168891	1.241968	H	-1.631705	1.387646	-1.816189	H	-1.631705	1.387646	-1.816189
H	-1.428628	-1.168891	-1.241968	F	0.578234	0.856730	-2.538423	F	0.578234	0.856730	-2.538423
F	-1.070557	0.884689	0.000000	O	0.125313	-1.094377	1.393805	O	0.125313	-1.094377	1.393805
N	-0.476986	-3.230946	0.000000	H	0.419834	-1.754182	2.028928	H	0.419834	-1.754182	2.028928
H	-1.403813	-3.641868	0.000000	N	-1.836325	0.876595	0.635796	N	-1.836325	0.876595	0.635796
H	0.005560	-3.590450	0.815961	H	-2.066021	1.818912	0.929899	H	-2.066021	1.818912	0.929899
H	0.005560	-3.590450	-0.815961	H	-1.362960	0.405620	1.400361	H	-1.362960	0.405620	1.400361
				H	-2.705165	0.386204	0.458505	H	-2.705165	0.386204	0.458505
1GeF:NH_3				1GeF$^{OH}_{rot}$:NH_3				1GeF^{OH}:NH_3			
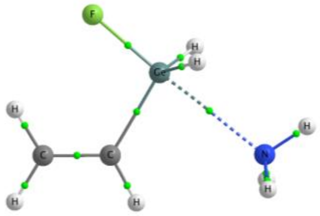				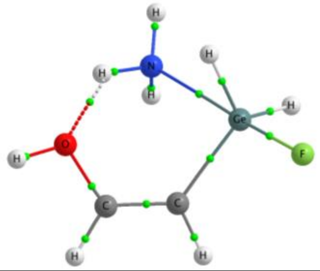				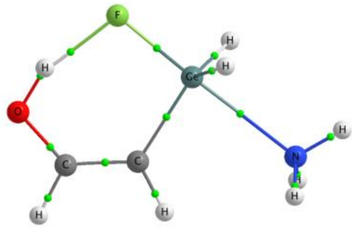			
C	1.137587	-0.725810	0.000000	C	-0.072184	-0.902637	-1.000469	C	0.441324	-1.315943	0.000000
C	1.843091	0.412264	0.000000	C	-0.149225	-1.922415	-0.144321	C	1.543107	-0.537696	0.000000
H	1.676384	-1.666027	0.000000	H	0.017129	-1.188062	-2.040998	H	0.648037	-2.377391	0.000000
H	2.926446	0.406588	0.000000	H	-0.143977	-2.960051	-0.460575	H	2.534815	-0.976591	0.000000
H	1.347378	1.374071	0.000000	Ge	0.071086	0.959657	-0.562366	Ge	-1.358963	-0.680711	0.000000
Ge	-0.783304	-0.692843	0.000000	H	0.429578	1.307359	0.862149	H	-2.162365	-0.739053	1.282689
H	-1.471834	-1.120749	1.278148	H	-0.867609	1.884472	-1.303070	H	-2.162365	-0.739053	-1.282689
H	-1.471834	-1.120749	-1.278148	F	1.590764	1.328044	-1.404951	F	-0.994005	1.097578	0.000000
F	-1.076447	1.051103	0.000000	O	-0.264331	-1.732034	1.214706	O	1.611287	0.802288	0.000000
N	-0.479783	-3.336546	0.000000	H	-0.169598	-2.581271	1.657076	H	0.694645	1.151101	0.000000
H	-1.401230	-3.759298	0.000000	N	-2.138399	0.498179	0.666110	N	-1.932111	-3.062485	0.000000
H	0.005324	-3.695690	0.814446	H	-2.530156	1.229219	1.247898	H	-2.936358	-3.202051	0.000000
H	0.005324	-3.695690	-0.814446	H	-1.892906	-0.282843	1.265244	H	-1.564239	-3.538169	0.816346
				H	-2.868732	0.183859	0.038566	H	-1.564239	-3.538169	-0.816346
1SiF:H_2O				1SiF$^{OH}_{rot}$:H_2O				1SiF^{OH}:H_2O			

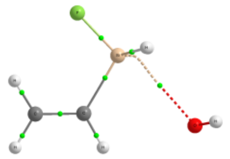

C	1.164324	-0.614442	0.000000
C	1.849890	0.538856	0.000000
H	1.714539	-1.548067	0.000000
H	2.933102	0.558435	0.000000
H	1.342285	1.495252	0.000000
Si	-0.694108	-0.631331	0.000000
H	-1.281684	-1.223956	1.217164
H	-1.281684	-1.223956	-1.217164
F	-1.154018	0.928213	0.000000
O	-0.082657	-3.450646	0.000000
H	-0.466730	-3.898473	0.760524
H	-0.466730	-3.898473	-0.760524

1GeF:H_2O

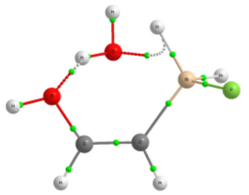

C	-0.341998	-1.041344	-1.012230
C	-0.133271	-1.653117	0.156149
H	-0.527004	-1.721638	-1.835935
H	-0.147981	-2.730999	0.272647
Si	-0.267314	0.777082	-1.394443
H	0.373129	1.569049	-0.331651
H	-1.553967	1.330669	-1.841986
F	0.712306	0.884603	-2.690115
O	0.096241	-0.953395	1.320934
H	0.300778	-1.575393	2.026374
O	-2.038492	0.840702	0.880040
H	-2.054937	1.699934	1.312051
H	-1.367185	0.335230	1.360494

1GeF$^{OH}_{rot}$:H_2O

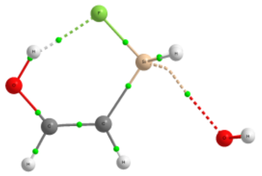

C	0.404531	-1.227901	0.017035
C	1.546410	-0.517978	-0.070368
H	0.537566	-2.300790	0.039376
H	2.515381	-1.000766	-0.114036
Si	-1.298927	-0.528139	0.098040
H	-2.017445	-0.750527	1.366380
H	-2.160864	-0.818744	-1.062577
F	-1.068377	1.103709	0.038645
O	1.692800	0.824645	-0.116773
H	0.815292	1.236607	-0.076466
O	-1.870564	-3.231157	0.207555
H	-2.432859	-3.530652	-0.514166
H	-2.343181	-3.488232	1.005867

1GeF^{OH}:H_2O

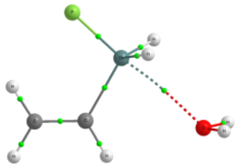

C	1.204582	-0.618342	0.000000
C	1.870219	0.543120	0.000000
H	1.757844	-1.549230	0.000000
H	2.952947	0.574280	0.000000
H	1.345772	1.489873	0.000000
Ge	-0.709505	-0.654455	0.000000
H	-1.342440	-1.197104	1.263846
H	-1.342440	-1.197104	-1.263846
F	-1.142808	1.045458	0.000000
O	-0.131034	-3.423606	0.000000
H	-0.440188	-3.924934	0.761206
H	-0.440188	-3.924934	-0.761206

1SiF:HCN

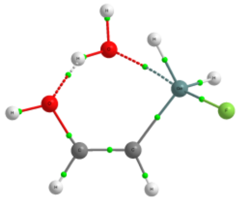

C	0.362749	-1.041942	0.685432
C	1.604569	-1.305114	0.280383
H	-0.004148	-1.688499	1.472242
H	2.210180	-2.098964	0.702337
Ge	-0.841000	0.262412	-0.030983
H	-0.488382	0.760085	-1.412082
H	-1.291900	1.313493	0.952836
F	-2.293741	-0.695865	-0.282511
O	2.229963	-0.561195	-0.698971
H	3.075373	-0.968717	-0.912298
O	1.380379	1.893337	0.351649
H	1.420531	2.705691	-0.162129
H	1.947368	1.267717	-0.123153

1SiF$^{OH}_{rot}$:HCN

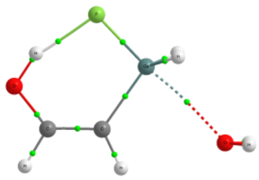

C	-0.531453	-1.108513	0.000000
C	0.313538	-2.155878	0.000000
H	-1.583933	-1.351431	0.000000
H	-0.054945	-3.175006	0.000000
Ge	0.000000	0.715137	0.000000
H	-0.262012	1.504792	1.264037
H	-0.262012	1.504792	-1.264037
F	1.776849	0.564209	0.000000
O	1.660831	-2.137601	0.000000
H	1.959937	-1.209408	0.000000
O	-2.698909	1.057108	0.000000
H	-3.088281	1.497154	0.762475
H	-3.088281	1.497154	-0.762475

1SiF^{OH}:HCN

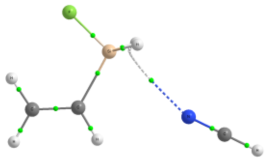

C	-1.257265	1.139026	0.000183
C	-2.457262	1.739471	-0.000271
H	-0.368940	1.759253	0.000580
H	-2.555653	2.818572	-0.000249
H	-3.374098	1.163490	-0.000671
Si	-1.109322	-0.717172	0.000145
H	-0.488852	-1.265669	1.219637
H	-0.488045	-1.265541	-1.219003
F	-2.643129	-1.266358	-0.000415
C	2.856907	0.039196	0.000249
N	1.688270	0.017263	0.000970
H	3.926743	0.057931	-0.000401

1GeF:HCN

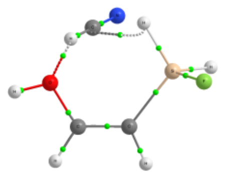

C	1.083283	0.802948	0.847343
C	0.289651	1.789977	0.417433
H	1.530764	0.983164	1.818242
H	0.119404	2.695474	0.992055
Si	1.522225	-0.753886	-0.067499
H	0.857179	-0.897866	-1.370191
H	1.383429	-1.947462	0.783134
F	3.115960	-0.645347	-0.388640
O	-0.406439	1.718235	-0.762795
H	-0.733049	2.596846	-0.981857
C	-2.360291	-0.833540	0.128987
N	-1.250994	-1.152001	0.317083
H	-3.365956	-0.517753	-0.054908

1GeF$^{OH}_{rot}$:HCN

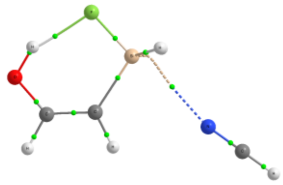

C	-0.853278	0.902146	0.000874
C	-2.052235	1.517679	0.000483
H	-0.006667	1.574352	0.001669
H	-2.137539	2.598000	0.000898
Si	-0.576228	-0.925555	0.000179
H	0.030038	-1.483431	1.220716
H	0.029718	-1.482609	-1.220905
F	-2.112987	-1.539460	0.000099
O	-3.280685	0.953419	-0.000609
H	-3.177385	-0.012004	-0.000831
C	3.186210	0.107927	-0.000617
N	2.037473	-0.105049	0.000128
H	4.238290	0.302932	-0.000613

1GeF^{OH}:HCN

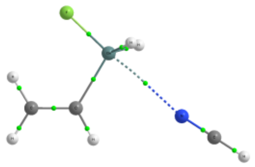

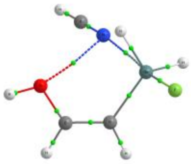

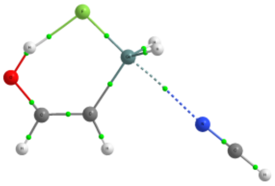

C	0.895532	1.374763	-0.000035
C	2.059772	2.035899	0.000019
H	-0.029845	1.937129	-0.000104
H	2.095955	3.118607	0.000054
H	3.003882	1.506639	0.000167
Ge	0.853802	-0.542561	0.000017
H	0.327922	-1.180075	-1.266831
H	0.328017	-1.179979	1.266961
F	2.563267	-0.956058	0.000000
C	-3.083135	0.044781	-0.000117
N	-1.916322	-0.017292	-0.000186
H	-4.151644	0.101253	0.000060

C	0.434301	0.819623	0.857481
C	-0.482837	1.686451	0.414345
H	0.818911	1.045674	1.845580
H	-0.803745	2.545863	0.995317
Ge	1.128254	-0.637742	-0.063308
H	0.533803	-0.853864	-1.389937
H	1.140977	-1.854529	0.765384
F	2.697682	-0.288844	-0.328014
O	-1.122592	1.534867	-0.789965
H	-1.568756	2.359532	-1.007874
C	-1.887041	-2.537922	0.589755
N	-0.860596	-2.008404	0.407113
H	-2.839599	-2.999683	0.745675

C	-0.677973	1.148464	-0.000265
C	-1.867325	1.779139	-0.000150
H	0.188719	1.792908	-0.000614
H	-1.928473	2.861364	-0.000375
Ge	-0.447025	-0.741658	-0.000074
H	0.078157	-1.376122	1.267515
H	0.077812	-1.376314	-1.267715
F	-2.156416	-1.276042	0.000196
O	-3.098960	1.232908	0.000366
H	-3.004532	0.261012	0.000666
C	3.294228	0.197967	-0.000079
N	2.152101	-0.045859	-0.000339
H	4.340457	0.422252	0.000245

1SiF:ACN | 1SiF$^{OH}_{rot}$:ACN | 1SiF^{OH}:ACN

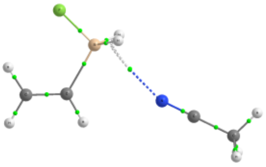

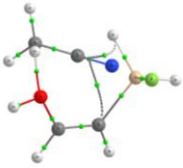

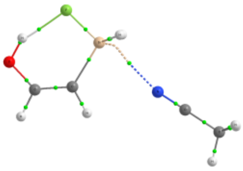

C	1.164905	-0.351913	-0.000838
C	1.868291	0.790791	-0.002116
H	1.704823	-1.291217	-0.001342
H	2.951862	0.794151	-0.003625
H	1.374950	1.754615	-0.001676
Si	-0.697113	-0.336472	0.001757
H	-1.296197	-0.906619	1.222013
H	-1.299585	-0.907074	-1.216625
F	-1.109647	1.239595	0.002014
C	-0.291556	-4.353757	0.000693
N	-0.210897	-3.187700	0.001473
C	-0.393872	-5.806434	-0.000261
H	-1.440329	-6.101533	-0.011642
H	0.099674	-6.210384	-0.880859
H	0.080284	-6.210167	0.891034

C	0.610610	0.291148	1.113261
C	0.618608	1.598646	0.826862
H	0.412811	0.042769	2.147853
H	0.455266	2.374679	1.567910
Si	0.954973	-1.040249	-0.135141
H	0.170814	-0.852343	-1.371923
H	0.726246	-2.368772	0.454620
F	2.513602	-0.968190	-0.572953
O	0.815052	2.041907	-0.454056
H	0.914225	2.999156	-0.451697
C	-2.373331	-0.530667	-0.005583
N	-2.254708	-1.517929	0.612537
C	-2.495337	0.698557	-0.775583
H	-1.504689	1.118492	-0.942442
H	-2.964120	0.490374	-1.734688
H	-3.103364	1.416975	-0.230141

C	0.537317	-0.985628	0.000890
C	1.669627	-0.254704	0.000499
H	0.696503	-2.054866	0.001685
H	2.648015	-0.720674	0.000914
Si	-1.184260	-0.312263	0.000195
H	-1.970448	-0.558620	1.220732
H	-1.969577	-0.558754	-1.220889
F	-0.948065	1.325638	0.000115
O	1.794757	1.091336	-0.000592
H	0.906900	1.484302	-0.000815
C	-2.169244	-4.087688	-0.000601
N	-1.779672	-2.986239	0.000144
C	-2.654774	-5.460238	-0.000596
H	-3.227488	-5.647737	0.904468
H	-3.291488	-5.625147	-0.866427
H	-1.813857	-6.148305	-0.039722

1GeF:ACN | 1GeF$^{OH}_{rot}$:ACN | 1GeF^{OH}:ACN

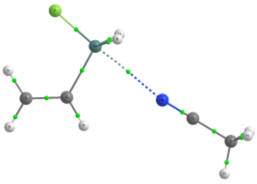

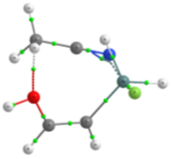

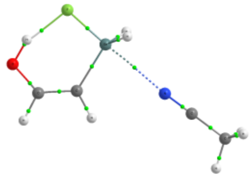

C	1.168513	-0.514612	0.004023
C	1.872839	0.624016	0.006789
H	1.695773	-1.460428	0.006935
H	2.956131	0.619570	0.011805
H	1.379365	1.587309	0.003949
Ge	-0.749006	-0.484410	-0.004978
H	-1.411691	-0.985498	1.258999
H	-1.399782	-0.986438	-1.274765
F	-1.098098	1.239358	-0.007289
C	-0.309730	-4.440604	-0.001187
N	-0.328000	-3.272284	-0.001678
C	-0.287469	-5.896355	-0.000731
H	-0.808714	-6.271921	0.876457
H	-0.777966	-6.271984	-0.895450

C	0.261434	0.628827	1.151611
C	0.072872	1.902718	0.795730
H	0.103905	0.395970	2.195225
H	-0.214101	2.679873	1.496652
Ge	0.857945	-0.712267	-0.074601
H	0.124843	-0.690269	-1.399367
H	0.921350	-2.076859	0.566290
F	2.509766	-0.305703	-0.481444
O	0.203884	2.301821	-0.507434
H	0.184681	3.262911	-0.553620
C	-2.435959	-0.760518	-0.013888
N	-2.124985	-1.744081	0.540194
C	-2.786591	0.468160	-0.709960
H	-1.873997	0.988651	-0.996773

C	-0.677973	1.148464	-0.000265
C	-1.867325	1.779139	-0.000150
H	0.188719	1.792908	-0.000614
H	-1.928473	2.861364	-0.000375
Ge	-0.447025	-0.741658	-0.000074
H	0.078157	-1.376122	1.267515
H	0.077812	-1.376314	-1.267715
F	-2.156416	-1.276042	0.000196
O	-3.098960	1.232908	0.000366
H	-3.004532	0.261012	0.000666
C	3.294228	0.197967	-0.000079
N	2.152101	-0.045859	-0.000339
C	4.717400	0.503059	0.000362
H	5.177202	0.109673	0.903689

H	0.740835	-6.249083	0.017120	H	-3.369917	0.238822	-1.598791	H	5.190359	0.050801	-0.867943
				H	-3.370906	1.109062	-0.053416	H	4.862308	1.580093	-0.034383
1SiCl:NH_3				1SiCl$^{OH}_{rot}$:NH_3				1SiCl^{OH}:NH_3			
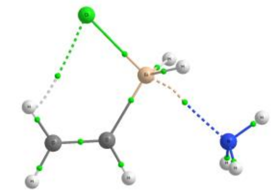				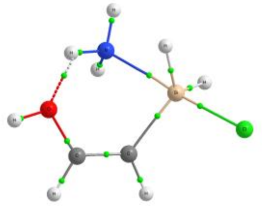				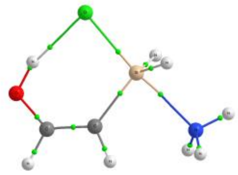			
C	1.095167	-0.731259	0.000000	C	-0.491842	-0.573575	-0.807380	C	-1.107255	-0.473659	-0.217195
C	1.849557	0.377623	0.000000	C	-1.004335	-1.613478	-0.142663	C	-0.971865	-1.801331	-0.004522
H	1.611438	-1.685059	0.000000	H	-0.199308	-0.797008	-1.826867	H	-1.981757	-0.241566	-0.815047
H	2.931781	0.322809	0.000000	H	-1.142985	-2.591506	-0.590638	H	-1.700965	-2.501397	-0.397573
H	1.402372	1.362959	0.000000	Si	-0.043061	1.112678	-0.138758	Si	-0.059849	0.936976	0.381482
Si	-0.774910	-0.711290	0.000000	H	0.211882	1.257371	1.304331	H	-0.205759	1.410444	1.771199
H	-1.418302	-1.189563	1.235305	H	-0.518691	2.287314	-0.891337	H	0.402384	1.929318	-0.605239
H	-1.418302	-1.189563	-1.235305	Cl	1.999197	1.146693	-0.828108	Cl	1.866472	-0.050791	0.678200
Cl	-1.271818	1.339364	0.000000	O	-1.403215	-1.520891	1.174691	O	-0.014988	-2.451506	0.676044
N	-0.442103	-3.329875	0.000000	H	-1.627560	-2.399945	1.495160	H	0.700262	-1.819852	0.887073
H	-1.374962	-3.726998	0.000000	N	-2.202859	1.187580	0.622380	N	-1.990426	2.154469	0.181112
H	0.032963	-3.700903	0.815281	H	-2.393526	2.007374	1.187663	H	-1.842856	3.102149	0.511978
H	0.032963	-3.700903	-0.815281	H	-2.355066	0.359931	1.191295	H	-2.722909	1.742815	0.749943
				H	-2.873673	1.171043	-0.137500	H	-2.341842	2.217595	-0.768501
1GeCl:NH_3				1GeCl$^{OH}_{rot}$:NH_3				1GeCl^{OH}:NH_3			
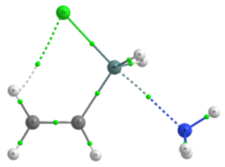				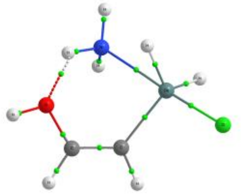				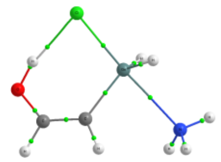			
C	1.121389	-0.706885	0.000000	C	-0.470510	-0.745006	-0.784396	C	-1.090792	-0.720888	-0.278839
C	1.871981	0.400688	0.000000	C	-1.081598	-1.749197	-0.153450	C	-0.963499	-2.026726	0.023447
H	1.620254	-1.668954	0.000000	H	-0.228328	-0.926821	-1.823323	H	-1.929840	-0.488648	-0.921258
H	2.953810	0.345494	0.000000	H	-1.342809	-2.680344	-0.644593	H	-1.679730	-2.755086	-0.339780
H	1.421524	1.384709	0.000000	Ge	0.164381	0.871955	0.023544	Ge	0.015633	0.716785	0.312731
Ge	-0.801114	-0.672801	0.000000	H	0.204729	0.913491	1.530148	H	-0.151130	1.209395	1.734029
H	-1.463693	-1.155850	1.270964	H	-0.188613	2.148395	-0.701784	H	0.384091	1.759185	-0.716434
H	-1.463693	-1.155850	-1.270964	Cl	2.318424	0.713119	-0.476771	Cl	2.011637	-0.292366	0.565079
Cl	-1.274890	1.467963	0.000000	O	-1.447636	-1.666228	1.170309	O	-0.016693	-2.620627	0.772053
N	-0.443996	-3.416230	0.000000	H	-1.740010	-2.533436	1.467962	H	0.712778	-1.988038	0.920292
H	-1.371552	-3.825371	0.000000	N	-2.349577	1.127008	0.648295	N	-2.184206	1.990444	0.182661
H	0.034571	-3.784990	0.814078	H	-2.629411	1.882750	1.262335	H	-2.114941	2.917009	0.588835
H	0.034571	-3.784990	-0.814078	H	-2.524949	0.251528	1.129980	H	-2.859297	1.472555	0.734530
				H	-2.954551	1.155024	-0.163705	H	-2.582219	2.103163	-0.743074
2T											
2SiF: NH_3				2SiF$^{OH}_{rot}$:NH_3				2SiF^{OH}:NH_3			
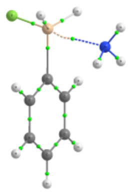				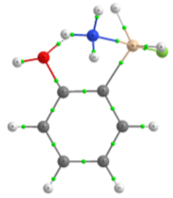				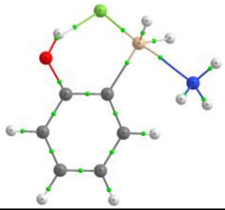			
C	2.170619	-0.133927	-1.206331	C	-2.153710	-1.404922	0.574309	C	2.139203	1.399535	-0.515103
C	2.869897	-0.121297	-0.000193	C	-2.885093	-0.304401	0.134583	C	2.886295	0.328034	-0.024876
C	2.170679	-0.139635	1.205906	C	-2.220922	0.814930	-0.360133	C	2.249798	-0.844831	0.360925
C	0.776402	-0.165945	1.202281	C	-0.828105	0.827991	-0.411301	C	0.859640	-0.952235	0.272411
C	0.053749	-0.174030	-0.000244	C	-0.061654	-0.259723	0.020018	C	0.074261	0.134173	-0.155150
C	0.776340	-0.160249	-1.202756	C	-0.762123	-1.374351	0.507072	C	0.753062	1.292166	-0.570398
H	2.709907	-0.125984	-2.145132	H	-2.661664	-2.280531	0.955744	H	2.630376	2.303809	-0.849220
H	3.952028	-0.101170	-0.000173	H	-3.966563	-0.311840	0.171310	H	3.964524	0.397717	0.038530
H	2.710018	-0.136116	2.144707	H	-2.783822	1.674943	-0.707580	H	2.810615	-1.699207	0.716614

H	0.246595	-0.183828	2.148908	Si	1.822818	-0.367677	-0.042190	Si	-1.803936	-0.002819	-0.167502
Si	-1.819091	-0.201536	-0.000282	H	2.442371	-0.876661	1.199145	H	-2.535786	0.759985	-1.195724
H	-2.418662	0.283723	-1.256660	H	2.565916	0.612622	-0.853377	H	-2.481852	-0.284621	1.115095
H	-2.418840	0.280005	1.257436	F	1.981238	-1.714858	-0.985744	F	-1.928999	-1.548609	-0.805756
F	-2.213560	-1.791069	-0.002601	N	1.679427	1.527332	1.306208	N	-1.773803	2.057715	0.856309
N	-1.293667	2.341226	0.003449	H	2.585912	1.902361	1.562292	H	-2.691137	2.259282	1.240986
H	-2.052739	3.013957	0.005220	H	1.182744	2.228649	0.765492	H	-1.119449	2.009348	1.630755
H	-0.717147	2.531824	0.815558	H	1.159122	1.361978	2.160345	H	-1.501529	2.852324	0.288800
H	-0.718747	2.534303	-0.809207	H	-0.200180	-2.244594	0.826726	H	0.185884	2.128017	-0.965672
H	0.246467	-0.173670	-2.149426	O	-0.151580	1.935084	-0.891429	O	0.321227	-2.150657	0.645532
				H	-0.796918	2.543857	-1.270403	H	-0.542261	-2.246374	0.211177
2GeF: NH_3				2GeF$^{OH}_{rot}$:NH_3				2GeF^{OH}:NH_3			
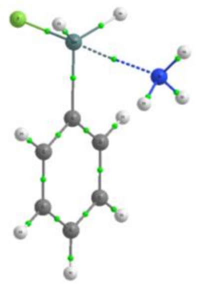				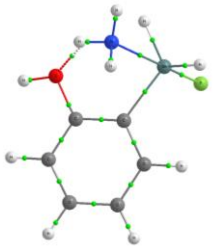				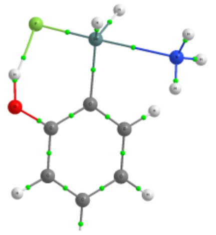			
C	2.529405	-0.194449	-1.206310	C	-2.411556	-1.433917	0.679483	C	2.428984	1.448802	-0.517979
C	3.227928	-0.206278	0.000046	C	-3.193207	-0.417907	0.135169	C	3.199491	0.400391	-0.014459
C	2.529476	-0.192758	1.206426	C	-2.584541	0.687945	-0.452382	C	2.592981	-0.783398	0.386149
C	1.135329	-0.164402	1.203391	C	-1.193979	0.771503	-0.495335	C	1.205515	-0.928318	0.300905
C	0.419715	-0.149468	0.000088	C	-0.385630	-0.232514	0.040819	C	0.406242	0.136416	-0.147164
C	1.135257	-0.166092	-1.203237	C	-1.022766	-1.333629	0.625921	C	1.046069	1.306729	-0.578023
H	3.068589	-0.210431	-2.144739	H	-2.878299	-2.297315	1.133924	H	2.898830	2.361746	-0.858050
H	4.309969	-0.228964	0.000029	H	-4.273064	-0.480650	0.163596	H	4.275763	0.496714	0.048482
H	3.068717	-0.207420	2.144845	H	-3.188484	1.482196	-0.877760	H	3.175184	-1.617748	0.754168
H	0.603608	-0.158512	2.148619	Ge	1.544914	-0.218084	-0.026840	Ge	-1.512438	-0.049641	-0.157375
Ge	-1.502963	-0.101139	0.000085	H	2.199670	-0.634503	1.272310	H	-2.270743	0.631906	-1.271564
H	-2.108706	0.388207	-1.296328	H	2.224373	0.824519	-0.878781	H	-2.213783	-0.265567	1.166249
H	-2.108716	0.389061	1.296159	F	1.815885	-1.685895	-0.988581	F	-1.574899	-1.769025	-0.714934
F	-1.961358	-1.806235	0.000618	N	1.157089	1.892759	1.356669	N	-1.518115	2.240095	0.809416
N	-0.808365	2.511487	-0.000998	H	1.959728	2.437098	1.650756	H	-2.339671	2.378151	1.388279
H	-1.472001	3.278262	-0.002617	H	0.608652	2.461017	0.719530	H	-0.707101	2.284094	1.417841
H	-0.211736	2.633175	0.809929	H	0.589096	1.717936	2.177450	H	-1.459149	3.035821	0.184015
H	-0.210284	2.630983	-0.811183	H	-0.417429	-2.136331	1.030888	H	0.452807	2.122617	-0.975508
H	0.603479	-0.161538	-2.148443	O	-0.563861	1.861454	-1.062469	O	0.687387	-2.126166	0.689144
				H	-1.227738	2.395695	-1.514825	H	-0.172497	-2.248340	0.242835
2SiCl: NH_3				2SiCl$^{OH}_{rot}$:NH_3				2SiCl^{OH}:NH_3			
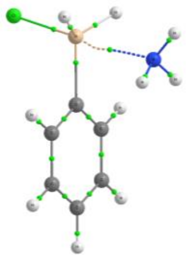				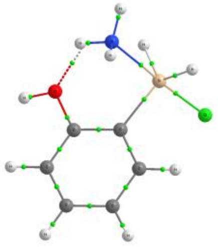				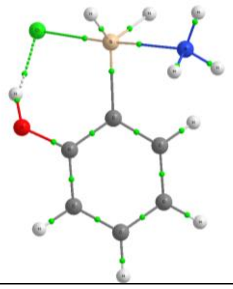			
C	-2.341600	-0.518262	1.206635	C	-1.977507	-1.694358	0.783166	C	2.577333	0.723009	-0.911252
C	-3.018967	-0.690996	0.000143	C	-2.925024	-0.959005	0.074342	C	3.068979	-0.394144	-0.234948
C	-2.341290	-0.519996	-1.206420	C	-2.543692	0.201992	-0.592619	C	2.201065	-1.224851	0.464117
C	-0.990925	-0.172605	-1.203978	C	-1.215794	0.622774	-0.545908	C	0.835031	-0.937840	0.505974
C	-0.295630	0.010812	-0.000016	C	-0.239108	-0.096643	0.150222	C	0.323139	0.213930	-0.115132
C	-0.991238	-0.170867	1.204030	C	-0.652685	-1.265106	0.807519	C	1.218789	1.016456	-0.841926
H	-2.862880	-0.657150	2.145307	H	-2.265128	-2.600072	1.299927	H	3.243668	1.354973	-1.483248
H	-4.066864	-0.961595	0.000199	H	-3.956230	-1.284389	0.034296	H	4.124579	-0.630637	-0.268627
H	-2.862328	-0.660235	-2.145025	H	-3.275072	0.778270	-1.149404	H	2.556705	-2.112570	0.970541
H	-0.474026	-0.049439	-2.149785	Si	1.579573	0.390098	0.237270	Si	-1.485406	0.688885	0.025609
Si	1.513697	0.478734	-0.000102	H	2.191877	0.274645	1.573621	H	-2.003132	1.614590	-0.995488
H	1.974236	1.099314	1.252356	H	2.111756	1.384583	-0.706134	H	-2.131070	0.645775	1.349320
H	1.974396	1.098579	-1.252861	Cl	2.436070	-1.379193	-0.632626	Cl	-2.376034	-1.153510	-0.714620

Cl	2.531168	-1.370479	0.000465	N	0.742409	2.317450	1.184274	N	-0.673150	2.622585	0.947351
N	0.312896	2.824335	-0.000863	H	1.463798	2.988460	1.424691	H	-1.410856	3.242500	1.267284
H	0.846272	3.686981	-0.001589	H	0.132307	2.737323	0.488988	H	-0.119711	2.360449	1.757153
H	-0.294471	2.842980	-0.812681	H	0.193436	2.138729	2.017896	H	-0.063077	3.156223	0.337569
H	-0.293725	2.843890	0.811490	H	0.087975	-1.860318	1.329225	H	0.841916	1.882826	-1.376996
H	-0.474579	-0.046321	2.149789	O	-0.817309	1.781823	-1.186885	O	0.044222	-1.808756	1.201293
				H	-1.547467	2.093017	-1.735401	H	-0.838505	-1.816252	0.789722
2GeCl: NH_3				$2GeCl^{OH}{}_{rot}$:NH_3				$2GeCl^{OH}$:NH_3			
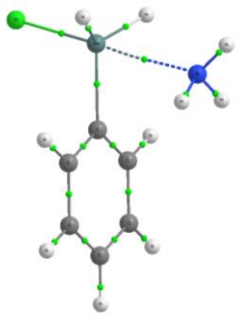				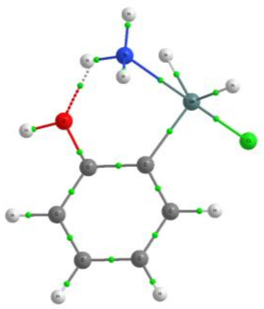				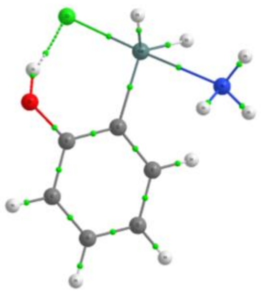			
C	-2.637042	-0.554527	1.206615	C	2.303836	-1.630786	-0.898869	C	2.802653	0.707423	-0.946966
C	-3.317302	-0.712328	-0.000079	C	3.215469	-0.948793	-0.096088	C	3.341668	-0.366976	-0.238395
C	-2.637504	-0.552370	-1.206747	C	2.790425	0.125066	0.681519	C	2.512440	-1.216842	0.484411
C	-1.280520	-0.231855	-1.204512	C	1.451725	0.512178	0.655135	C	1.135103	-0.990151	0.518693
C	-0.587142	-0.066841	-0.000023	C	0.518070	-0.157228	-0.137497	C	0.581959	0.116245	-0.141348
C	-1.280058	-0.234007	1.204437	C	0.968555	-1.233936	-0.909812	C	1.431462	0.939259	-0.892664
H	-3.160757	-0.683846	2.145007	H	2.627643	-2.468159	-1.501955	H	3.442345	1.354433	-1.531881
H	-4.370281	-0.962297	-0.000099	H	4.254864	-1.248347	-0.069737	H	4.407067	-0.555896	-0.264243
H	-3.161575	-0.680019	-2.145172	H	3.496224	0.659184	1.308390	H	2.908119	-2.071400	1.017004
H	-0.760228	-0.115249	-2.148871	Ge	-1.356208	0.298363	-0.180529	Ge	-1.295022	0.507622	-0.008607
Ge	1.282060	0.379639	0.000036	H	-1.941003	0.288923	-1.574321	H	-1.842484	1.429672	-1.070502
H	1.755511	1.010407	1.289096	H	-1.859818	1.333301	0.791127	H	-1.904808	0.563217	1.372585
H	1.755360	1.011108	-1.288735	Cl	-2.235227	-1.560759	0.639666	Cl	-2.144776	-1.470159	-0.644261
Cl	2.297058	-1.564998	-0.000490	N	-0.322215	2.480959	-1.127175	N	-0.397690	2.712448	0.917693
N	-0.016300	2.827143	0.000862	H	-0.928049	3.252282	-1.382502	H	-1.072970	3.281401	1.417278
H	0.426043	3.739534	0.001607	H	0.251022	2.780032	-0.345123	H	0.301277	2.414118	1.590180
H	-0.624339	2.789345	-0.809759	H	0.297043	2.307381	-1.910393	H	0.078625	3.320773	0.261186
H	-0.624734	2.788206	0.811133	H	0.255016	-1.783182	-1.513373	H	1.011688	1.773981	-1.443904
H	-0.759407	-0.119108	2.148806	O	1.001033	1.581156	1.402153	O	0.378261	-1.868418	1.240934
				H	1.703318	1.848894	2.007175	H	-0.500953	-1.935064	0.826433

Table S2. Molecular electrostatic potential maxima corresponding to the σ-hole on the tetrel atom, in kcal/mol, on the 0.001 a.u. electron density isosuface at the MP2/aug-cc-pVTZ computational level.

	F	Cl
1SiX:NH_3	0.0156	0.0150
$1SiX^{OH}$:NH_3	0.0173	0.0161
$1SiX^{OH}{}_{rot}$:NH_3	0.0088	0.0087
1GeX: NH_3	0.0183	0.0165
$1GeX^{OH}$:NH_3	0.0203	0.0176
$1GeX^{OH}{}_{rot}$:NH_3	0.0115	-
2SiX:NH_3	0.0108	0.0093
$2SiX^{OH}$:NH_3	0.0148	-

$2SiX^{OH}{}_{rot}:NH_3$	0.0093	0.0114
$2GeX: NH_3$	0.0136	0.0105
$2GeX^{OH}:NH_3$	0.0169	0.0130
$2GeX^{OH}{}_{rot}:NH_3$	0.0123	0.0094

Table S3. Electron density, Laplacian, and total energy density, H at the bond critical point, in a.u. and intermolecular distance, in Å, at the MP2/aug-cc-pVTZ computational level.

Complex	ρ_{BCP}	$\nabla^2\rho_{BCP}$	H_{BCP}	Distance
$1SiF:NH_3$	0.0270	0.0489	-0.0050	2.518
$1SiF^{OH}:NH_3$	0.0405	0.0817	-0.0135	2.276
$1SiF^{OH}{}_{rot}:NH_3$	0.0372	0.0626	-0.0119	2.335
$1GeF: NH_3$	0.0241	0.0641	-0.0009	2.661
$1GeF^{OH}:NH_3$	0.0366	0.0916	-0.0051	2.450
$1GeF^{OH}{}_{rot}:NH_3$	0.0289	0.0742	-0.0022	2.570
$1SiCl:NH_3$	0.0224	0.0452	-0.0024	2.640
$1SiCl^{OH}:NH_3$	0.0401	0.0718	-0.0135	2.291
$1SiCl^{OH}{}_{rot}:NH_3$	0.0406	0.0707	-0.0140	2.291
$1GeCl: NH_3$	0.0199	0.0539	0.0000	2.767
$1GeCl^{OH}:NH_3$	0.0305	0.0769	-0.0028	2.545
$1GeCl^{OH}{}_{rot}:NH_3$	0.0274	0.0696	-0.0018	2.603
$1SiF:H_2O$	0.0117	0.0364	0.0006	2.885
$1SiF^{OH}:H_2O$	0.0142	0.0417	0.0002	2.765
$1SiF^{OH}{}_{rot}:H_2O$	0.0125	0.0364	0.0005	2.883
$1GeF:H_2O$	0.0137	0.0474	0.0012	2.829
$1GeF^{OH}:H_2O$	0.0166	0.0572	0.0011	2.720
$1GeF^{OH}{}_{rot}:H_2O$	0.0158	0.0509	0.0009	2.782
1SiF:HCN	0.0101	0.0336	0.0010	2.991
$1SiF^{OH}:HCN$	0.0123	0.0386	0.0007	2.873
$1SiF^{OH}{}_{rot}:HCN$	-	-	-	-
1GeF:HCN	0.0129	0.0449	0.0013	2.898
$1GeF^{OH}:HCN$	0.0158	0.0536	0.0011	2.789
$1GeF^{OH}{}_{rot}:ACN$	0.0132	0.0458	0.0013	2.891

2T				
Complex	ρ_{BCP}	$\nabla^2\rho_{BCP}$	H_{BCP}	Distance
2SiF:NH_3	0.0241	0.0468	-0.0033	2.596
2SiF^{OH}:NH_3	0.0396	0.0665	-0.0135	2.301
2SiF$^{OH}{}_{rot}$:NH_3	0.0377	0.0641	-0.0122	2.330
2GeF: NH_3	0.0224	0.0602	-0.0005	2.703
2GeF^{OH}:NH_3	0.0301	0.0762	-0.0026	2.485
2GeF$^{OH}{}_{rot}$:NH_3	0.0345	0.0843	-0.0043	2.553
2SiCl:NH_3	0.0229	0.0452	-0.0026	2.635
2SiCl^{OH}:NH_3	0.0408	0.0691	-0.0141	2.291
2SiCl$^{OH}{}_{rot}$:NH_3	0.0399	0.0666	-0.0136	2.305
2GeCl: NH_3	0.0200	0.0539	0.0000	2.771
2GeCl^{OH}:NH_3	0.0303	0.0749	-0.0028	2.554
2GeCl$^{OH}{}_{rot}$:NH_3	0.0280	0.0708	-0.0020	2.594
